# Knowledge, Attitudes, Practices and Risk Perceptions of Antimicrobial Use and Resistance Among Small-Scale Fish Farmers in Cameroon

**DOI:** 10.1155/vmi/2042800

**Published:** 2025-10-12

**Authors:** Mohamed Moustapha Fokom Ndebé, Frédéric Moffo, Jenny Maïva Yango Tchoumbougnang, Mildred Naku Tangu, Cyrille Talla Ngueko, Julius Awah-Ndukum, Mohamed Moctar Mouliom Mouiche

**Affiliations:** ^1^Department of Animal Science, Laboratory of Animal Physiology and Health, University of Dschang, BP 222, Dschang, West Region, Cameroon; ^2^Institute of Agricultural Research for Development, Bangangté Polyvalent Station, BP 222, Bangangté, West Region, Cameroon; ^3^Department of Pharmacy, Pharmacology and Toxicology, University of Ngaoundéré, BP 454, Ngaoundéré, Adamawa Region, Cameroon; ^4^One Health Innovative Solutions (OHIS) Research Unit, University of Ngaoundéré, BP 454, Ngaoundéré, Adamawa Region, Cameroon; ^5^Department of Animal Production Technology, University of Bamenda, BP 39, Bambili, Northwest Region, Cameroon

**Keywords:** antimicrobial resistance, antimicrobial use, attitude, Cameroon, fish farmers, knowledge, practice, risk perception

## Abstract

Antimicrobial resistance (AMR) is a complex public health problem that is caused mainly by the inappropriate use of antimicrobial agents. The rational use of antimicrobial agents, which is the main strategy for preventing AMR, can be achieved through farmers' knowledge and behaviour. Hence, this study aimed to assess the knowledge, attitudes and practices of fish farmers regarding antimicrobial use (AMU), AMR and their perceptions (KAPP) of the risk of the emergence of AMR in Cameroon. A cross-sectional study of 364 fish farmers was performed in the Adamawa, Centre, Littoral, South and West Regions using a questionnaire. Descriptive statistics were used to summarise the data. To compare group differences, the Kruskal–Wallis and Wilcoxon tests were run at a significance level of 0.05. Pearson correlation and linear regression were used to determine the directionality, magnitude and nature of the relationships among variables. Overall, the survey revealed poor knowledge of AMU (0.47 ± 0.19) and AMR (0.30 ± 0.29). The attitudes towards and practices of AMU were also poor (0.40 ± 0.28 and 0.47 ± 0.11, respectively). However, the level of risk perception of the emergence of AMR by the fish farmers was fair (0.50 ± 0.29). Locality, age, professional experience, educational level of the fish farmer, training in fish farming and presence of health agents on the farm were associated (*p* < 0.05) with KAPP measures. Thus, training and updating fish farmers on modern fish farming practices with an emphasis on knowledge of the rational use of antimicrobials will have a positive impact on appropriate attitudes, best AMU practices and, consequently, a better perception of the risk of AMR emergence and the risk to public health.

## 1. Introduction

Fish are important sources of protein, vitamins, minerals and oils with low levels of cholesterol and are reasonably expensive [1]. The exponential worldwide development of fish farming has been accompanied by an increase in antimicrobial use (AMU), which is critically important for human health [2]. Antimicrobials are used in fish farming for therapeutic and nontherapeutic purposes [3] and as growth promoters and for increasing fish reproduction and tranquilisation [4]. Global antimicrobial consumption in aquaculture is rapidly increasing with the industrialisation of the sector. In 2017, the global antimicrobial consumption was estimated to be 10259 tons, increasing by 33% to 13600 tons in 2030 [5]. The use of antimicrobials in the treatment and prevention of diseases has been considered a solution to limit economic losses [6, 7]. However, in most African countries, the choice of antimicrobial agent is not usually based on knowledge of bacterial susceptibility [8]. In Cameroon, the total import of antimicrobial agents for use in food-producing animals increased by 104% between 2014 and 2019 [9]. On average, 143.8 mg (ranging from 0.5 to 2061.79 mg/kg) of antimicrobial agent was reported to produce 1 kg of chicken [10]. Fish farmers commonly practice earthen fish ponds or integrate fish farming systems with poultry, piggery and crop farming [11]. This system is mainly associated with antimicrobials used to alleviate hygiene adulteration. Additionally, livestock manure used for fish pond fertilisation might be contaminated by antimicrobial residue and resistant bacteria [12, 13]. Tetracycline, penicillin, sulfamethazole, virginiomycin, erythromycin, enrofloxacin and chloramphenicol are mostly used in fish farms [14, 15]. Chemical substances such as disinfectants and biocides used to ensure good water quality together with antimicrobial residues from integrated production systems may contribute to the selectivity, emergence and spread of drug-resistant pathogens, which pose serious threats to public health [16].

Though data are limited, there is increasing concern about antimicrobial resistance (AMR) in aquaculture which may be an underestimated route for transmission of resistant bacteria from aquatic environments to humans [5]. Aquaculture systems are major hotspots for AMR, promoting genetic exchange, and the use of antibiotics may promote development of pathogenic and antimicrobial-resistant bacteria in fish farms [6]. About 90% of aquatic bacteria are resistant to at least one antibiotic, 20% of them being resistant to five or more antibiotics, and can persist in the environment, even without selective pressure [7]. There are high levels of multidrug resistance of aquaculture bacteria to commonly used antibiotics such as tetracycline and ampicillin in Africa, with high multidrug resistance rates observed in *E. coli* (43.1%), *Salmonella* spp. (40.3%) and *Staphylococcus* spp. (31.3%) [17]. Therefore, strengthening regulations on the use of veterinary drugs would be beneficial in most African countries. Though specific regulations on the use of antimicrobials is lacking in Cameroon, a general framework governs veterinary medicines, and a management plan for antiparasitic and antibiotic use has been developed in the country. However, there are no targeted measures to limit the use of critically important antimicrobials, and aquaculture is not included in the multisectoral action plan for proper antimicrobial management.

Raising awareness among livestock farmers through educational campaigns and increased veterinary assistance could not only encourage them to adopt more prudent and rational practices but also possibly reduce the use of antimicrobial agents [18]. Education is a key element that changes farmers' behaviour towards AMU. Previous studies have indicated that farmers with postgraduate degrees better manage antimicrobial treatment than uneducated farmers [19]. There is a need for essential insights to recommend interventions for responsible management of antimicrobials in aquaculture in many African countries in Cameroon where there is growing interest in fish farming.

Despite the decrease in the use of antimicrobial agents during recent decades, partly due to the ban on growth-promoting treatments in many countries, information on AMU is scarce in low- and middle-income countries, hindering assessments of human, animal and environmental risks [20–24].

Therefore, this study was carried out to evaluate the knowledge, attitudes, practices and risk perceptions related to AMU and AMR among small-scale fish farmers in five regions of Cameroon.

## 2. Materials and Methods

### 2.1. Study Area

Table 1 outlines the main demographic and geographic features of the study areas.

**Figure 1:**
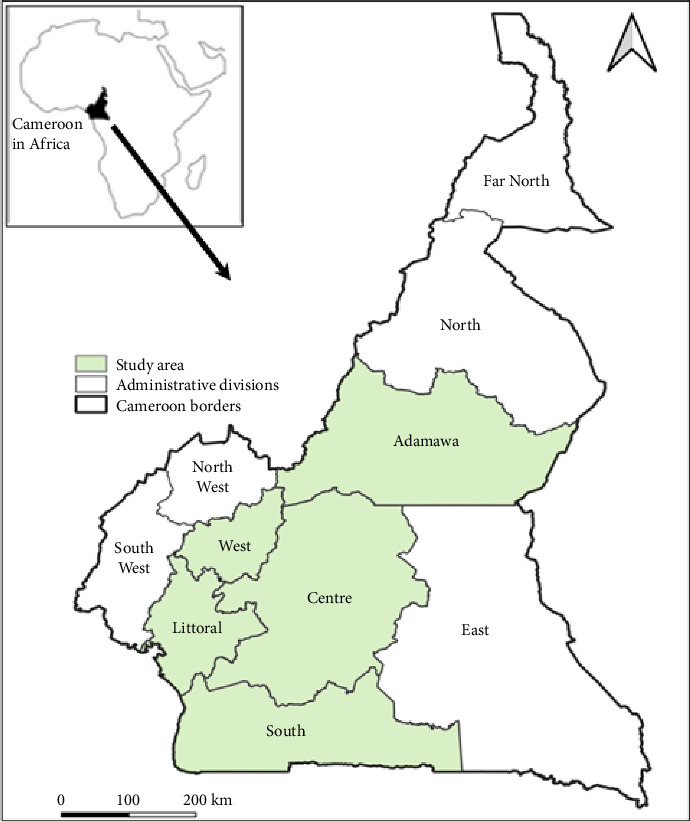
Map showing administrative regions including the study regions (Adamawa, Centre, Littoral, South and West) of Cameroon.

These five regions contribute to more than 72.48% of the total fish production in Cameroon [25].  Figure 1 shows location of the different study regions in Cameroon.

### 2.2. Study Design and Population

This cross-sectional indirect survey was conducted from March to September 2021. The target population was all fish farmers who were active at the time of the survey, regardless of the farming system, the species farmed or even the farming method.

### 2.3. Sample Size and Sampling Method

A minimum sample size of 384 was estimated using the appropriate tools (https://epitools.ausvet.com.au/content.php?page=1Proportion), based on a prevalence of 50%, a confidence interval of 95% and a precision of 5%. The Scientific Research and Ethics Committee of the School of Veterinary Medicine and Sciences (Ref No: 2021/0001/UN/ESMV/DAACRS/SSFC) granted ethical permission for this cross-sectional study in five regions of Cameroon. Ten farmers were chosen at random from the research regions to assess the quality, reliability and fluidity of the questionnaire. The reliability of the questionnaire was assessed using McDonald's Omega (*ω*) and was considered reliable when *ω* was ≥ 0.80. The regional delegations in charge of animal health permitted the survey in five regions (Adamawa (Ref No: 000042/21/L/RA/DREPIA/SRDPIA), Centre (Ref No: 000130/L/MINEPIA/SG/DREPIA-CE), Littoral (Ref No: 066/L/R-LT/RDREPIA/SRSV), South (Ref No: 031/L/R-SU/DREPIA/SRSV) and West (Ref No: 022/21/L/DREPIA-O/SRAG)). The selection of fish farms was performed using a random number generation technique from a list of fish farmers obtained at the Delegations of Livestock, Fisheries and Animal Industries (DREPIA) in the study regions and completed by private field veterinary practitioners. The purpose of the study was explained (with the assistance of local veterinary and fisheries practitioners, community leaders and trusted intermediaries) to fish farmers in the selected regions. The active and adult fish farmers were included in the study after their individual informed verbal consent was obtained. Completing the questionnaire further implied consent to participate in the study. Face-to-face interviews and on-farm observations were used to complete the questionnaire in order to evaluate the KAPP measures.

### 2.4. Questionnaire Design and Data Collection

The study was conducted using a questionnaire designed based on previous studies reported in the literature [14, 16, 26, 27]. The questionnaire used for the survey was divided into five parts: (i) demographic characteristics of the fish farmers (gender, age, education, training and experience in fish farming), (ii) farming characteristics (farm type, stocking density and husbandry system), (iii) fish farmer knowledge about AMU and AMR, (iv) attitudes and practices related to AMU and (v) how fish farmers perceive the risk of developing AMR. The responses included dichotomous and categorical outcomes (yes/no; a/b/c/d), ordinal results (5-point Likert scale type; strongly agree/agree/neutral/disagree/do not know) and open-ended questions.

The questionnaire was administered in French by three young veterinarians. Before starting the field work, they were trained on how to administer the questionnaire. The main objectives of the project were discussed with the farmers, and their consent was obtained before the questionnaire was administered.

### 2.5. Data Management and Statistical Analysis

Responses from the field were recorded into binary categories with 1 denoting sufficient knowledge, a desirable attitude, an appropriate behaviour regarding the use and resistance of antibiotics and a positive perception of the risk of such resistance and 0 denoting insufficient knowledge, an unfavourable attitude, improper behaviour and a negative perception of risk. At the attitude, practice and perception levels, responses such as ‘indifferent', ‘sometimes' and ‘do not know' were categorised as unwanted, improper and unfavourable, respectively. For the open-ended questions, adequate replies were categorised as 1, and insufficient replies were categorised as 0 [28].

The sum of the good responses recorded for each observation was divided by the maximum score to obtain a proportion of correct answers. Maximum score = sum of the highest score for each question obtainable by the farmers [29]. The scores obtained from the KAP questionnaires were calculated and defined as ‘good' (≥ 75%), ‘fair' (50%–75%) or ‘poor' (< 50%) as previously described by Mateo et al. [29]. Descriptive statistics were used to indicate the demographic distribution of the farmers and farm characteristics. Kruskal–Wallis and Wilcoxon tests were performed to compare mean scores between variables at the 95% confidence level. The normality of the residuals was tested with the Shapiro–Wilk test. Dunn's post hoc pairwise comparison test was also performed to assess significant differences in the mean KAPP score across regions. Pearson correlation tests were also performed to assess the relationships between mean KAPP scores across and within regions. Independent *t* tests were used to compare dichotomous variables. The relationships between demographic and farm characteristics and KAPP scale scores were explored using a linear regression model. Data entry and analysis were performed using Microsoft Excel 2013 (Microsoft Corporation, Redmond, WA, USA) and R (Vers. 4.1.2).

## 3. Results

### 3.1. Demographic and Farm Characteristics of the Fish Farms Surveyed in Cameroon

A total of 386 questionnaires were completed during the investigation, 364 (94.3%) of which were included in the analysis. Most of the fish farmers investigated during the study period were males (75.00%) and aged between 36 and 50 years (40.93%). More than half of the respondents had a higher level of education (59.34%) and had training in fish farming (82.14%). More than half of the respondents (70.89%) had less than 5 years of fish production. They have semi-intensive units, and raising fish requires less than 8 months per production cycle. Most farms had fair biosecurity levels (Table 2).

**Figure 2:**
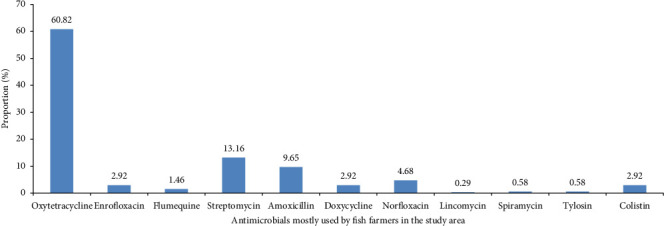
Proportion of antimicrobial agents mostly used by fish farms in the Adamawa, Centre, Littoral, South and West regions of Cameroon.

For antibiotic treatment, the farmers surveyed in the study area mostly used oxytetracycline (60.82%), streptomycin (13.16%) and amoxicillin (9.65%) (Figure 2).

### 3.2. Knowledge of AMU and AMR

Among the 364 respondents, 320 (87.91%) had already heard about antimicrobial agents, and 224/320 (70%) knew that antimicrobial agents act on bacteria. However, 24.69% were unable to correctly define antimicrobial agents (Table 3).

**Figure 3:**
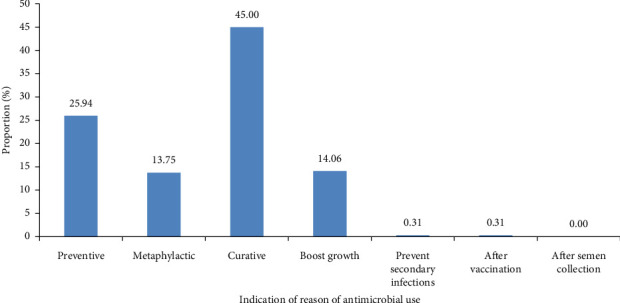
Reasons for the use of antimicrobials by fish farmers (*n* = 320) in the Adamawa, Centre, Littoral, South and West regions of Cameroon.

For reasons related to the use of antimicrobials, less than half of the respondents (45%) applied them for curative purposes, while about a quarter (25.94%) of them used them for preventive treatment (Figure 3).

**Figure 4:**
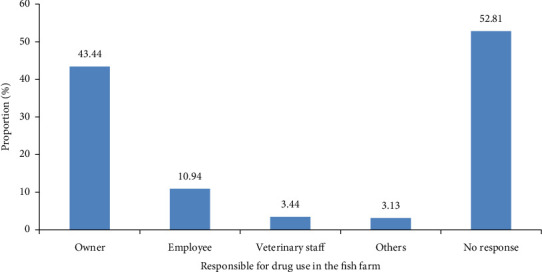
Person responsible for drug administration in fish farms (*n* = 320) in the Adamawa, Centre, Littoral, South and West regions of Cameroon.

Antimicrobials were mostly applied on farms by farm owners (43.44%) (Figure 4).

**Figure 5:**
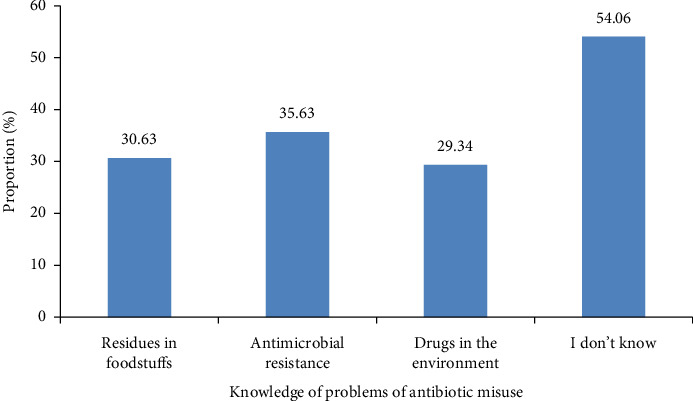
Knowledge of the problems associated with the misuse of antibiotics in the Adamawa, Centre, Littoral, South and West regions of Cameroon.

Approximately half of the fish farmers had no knowledge of the problems associated with the misuse of antibiotics (54.06%). However, the presence of drugs in the environment (29.34%) is less known by farmers as a problem of antibiotic misuse (Figure 5).

**Figure 6:**
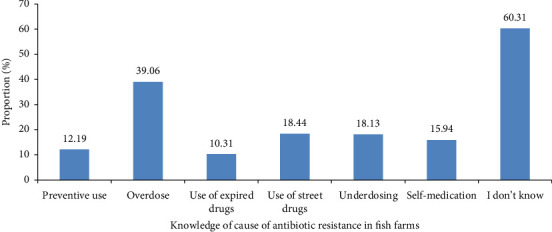
Knowledge about antibiotic use that can lead to antibiotic resistance in fish farms (*n* = 320) in the Adamawa, Centre, Littoral, South and West regions of Cameroon.

Additionally, 60.31% of the fish farmers had no knowledge of practices that can lead to AMR. The other respondents mostly mentioned overdose (39.06%), and the use of expired drugs was very low (10.31%) (Figure 6).

Overall, the mean AMU and AMR knowledge scores of the fish farmers in the study area were 0.47 ± 0.19 and 0.30 ± 0.29, respectively.

The mean AMU (0.53 ± 0.18) and AMR (0.44 ± 0.32) scores were significantly (*p* < 0.05) greater in the Littoral region than in the other regions. The level of education, training in fish farming and consultation with animal health professionals influenced farmers' knowledge of AMU and AMR (Table 4).

### 3.3. Practices and Attitudes Towards AMU and Risk Perceptions of AMR Among Fish Farmers

**Figure 7:**
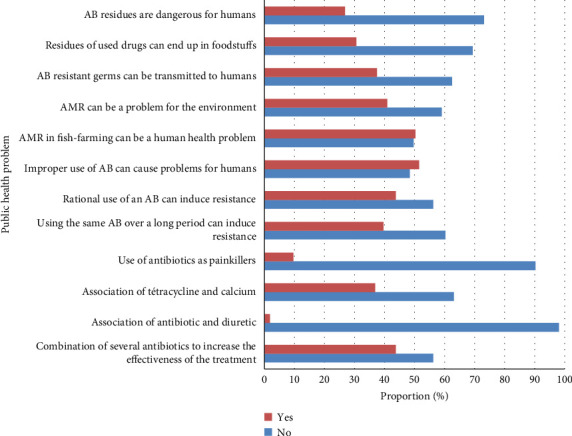
Inappropriate practices adopted by fish farms and knowledge of antibiotic use practices are likely to create a public health problem in the Adamawa, Centre, Littoral, South and West regions of Cameroon. Legend: AB = antibiotics; AMR = antimicrobial resistance.

Most respondents were aware of the prudent use of antimicrobials on fish farms; few of them combined tetracycline with minerals or diuretics during drug administration (Figure 7).

Fewer than half of the farmers adopted appropriate practices (0.47 ± 0.11) for AMU, with significantly greater scores in the Centre (0.50 ± 0.10) region than in the other regions. With respect to the attitudes concerning AMU, fish farmers with higher levels of education (0.46 ± 0.28) adopted more desirable attitudes than others did. The mean risk perception of AMR was 0.50 ± 0.29. However, region, level of education, training in fish farming and experience significantly influenced fish farmers' perceptions of the risk of AMR (Table 5).

### 3.4. Associations Between Knowledge, Attitudes, Practices and Risk Perception Scale Scores Across Regions

Globally, KAPP across regions was significantly (*p* < 0.05) and positively correlated with a strong correlation between AMU knowledge and AMR knowledge (*r* = 0.63) and attitudes (*r* = 0.62). Knowledge of AMR was strongly associated with attitudes (*r* = 0.72) and perceptions (*r* = 0.55). There was also a strong correlation between attitudes towards and risk perceptions of AMR (*r* = 0.60). Strong positive correlations were observed between KAPP scale scores within the region, ranging from 0.50 for attitudes towards knowledge of AMU in the South Region to 0.85 for risk perception of AMR. However, there were weak nonsignificant correlations between practices and knowledge of AMU (*r* = −0.07), knowledge of AMR (*r* = −0.28) and attitudes (*r* = −0.14) in the South region. The same was also observed between biosecurity and the risk perception of AMR (*r* = −0.08) in the Western region (Table 6).

### 3.5. KAPP Scale Measures and Demographic or Farm Dynamics Characteristics

Some demographic and farm characteristics were significantly correlated with the KAPP measures. Region was positively associated with AMU knowledge (a 1% increase), while age was negatively associated with AMU knowledge (a 2% decrease) and practices (a 4% decrease). Experience in fish farming was negatively associated with practices (2% decrease) and perceptions (7% decrease). Educational level was negatively associated with knowledge of AMU (2% decrease) and attitudes (5%). Training in fish farming was positively associated with knowledge of AMU (8% increase), knowledge of AMR (11% increase), attitudes (12% increase) and perceptions (9% increase). The presence of a health agent was positively associated with knowledge of AMU (3% increase), attitudes (6% increase) and practices (4% increase) (Table 7).

Overall, fish farmers showed poor levels of knowledge of AMU (73.13%), AMR (72.19%) and attitudes towards antimicrobials (63.75%), practices (79.38%) and perceptions (65.94%) of the risk of AMR (Table 8).

## 4. Discussion

Knowledge of the factors that encourage the use of drugs is essential for understanding and preventing the risk of AMR to public health. Consequently, reducing this risk requires the intervention of an essential link in the use of drugs, which is the farmer [26]. The results of this study suggest that a poor level of knowledge of AMU and AMR is associated with the level of education and lack of awareness of AMU and AMR. This result contrasts with the high percentage of sufficient knowledge (88.89%) obtained by Waga et al. in 2022 in Kenya [30]. Indeed, the level of knowledge of AMU and AMR increases with educational level. Despite this, respondents who had gone through university barely received a fair knowledge score on the AMU and a poor knowledge score on the AMR. This is an indication that limited formal education and lack of access to proper training and extension services on responsible AMU might hamper the full understanding of the concepts of AMR and the importance of judicious usage. This aligns with the findings of Moffo et al. [26] in Cameroon, following a KAPP study conducted in the poultry sector. Consequently, it would be wise to combine education with awareness-raising and education mechanisms for fish farmers on the use of antimicrobials and AMR to achieve an acceptable level of knowledge of AMU and AMR. This finding corroborates Pham-Duc et al. [31], who showed that farmers with a high level of education have a significantly greater level of knowledge and skills than others. In contrast, Ndashe et al. [32] in Zambia, following a KAP study in aquaculture, reported that while formal education was important, specialised training significantly influenced the knowledge of farmers on AMU best practices and AMR.

The lack of health workers on fish farms and the lack of training in fish farming and fish farming as a secondary activity are other explanations for the low level of knowledge of AMU and AMR. As a result, farms with a health worker, farms whose farmers are trained in fish farming and those with fish farming as their main activity had a significantly better level of knowledge of AMU and AMR than the other farms did. Moreover, nearly two-thirds of the respondents had no knowledge of antibiotic use behaviour that can lead to antibiotic resistance. Hence, there is a strong positive correlation between AMU knowledge and AMR knowledge. However, knowledge and breeder behaviour can influence AMU on farms [18]. Many fish farmers have already heard about antibiotics, which corroborates Waga et al. [30], who reported that 94.44% of respondents were aware of antibiotics in Kenya. These findings are also comparable to those of Pham-Ducet al. [31] in Vietnam, which showed high familiarity (80.4%) with antibiotics by livestock and aquaculture producers [31]. Accessible, farmer-friendly educational materials and awareness campaigns that effectively communicate the risks of AMR in aquaculture are needed to increase awareness among farmers and empower them with disease prevention and management strategies other than the use of antimicrobial agents to address hygiene adulteration.

In Cameroon, fish farmers use antibiotics for preventive, metaphylactic and curative reasons to boost growth and prevent secondary infections. Even if the first reason is curative, which corroborates Pham-Duc et al. [31], many fish farmers in Cameroon do not know the germ on which the antibiotic is effective, hence constituting a major concern about AMR. In most African countries, the choice of antimicrobial agent is not usually based on knowledge of bacterial susceptibility [8]. This favours the use of very broad-spectrum antibiotics such as oxytetracycline, which are extensively used by the fish farmers in this study. This high use of oxytetracycline in Cameroonian aquaculture is also observed in 73% of the 15 largest aquaculture producers [33]. However, in Cameroon, many users of tetracycline in fish farming combine it with calcium. This combination, which leads to changes in the conformation of tetracycline by chelation and the formation of a ternary complex, can have a significant effect on the bioavailability and antimicrobial activity of antibiotics [34].

In fact, the use of nonselective AMU to treat and prevent diseases is common in aquaculture and can lead to the emergence of antimicrobial-resistant germs, which potentially impact public health and entire ecosystems [35]. In addition, bacteria from fish, especially Enterobacteriaceae, can exchange resistance genes with humans [36], decreasing their therapeutic potential in animals and humans. This attitude can be justified by the fact that more than half of the fish farmers have no knowledge of the problems linked to the misuse of antibiotics. Moreover, if some fish farmers are aware that antibiotics are effective against bacteria, the problem lies in the rationality of their use. Indeed, approximately half of the respondents do not know that the long-term use of antibiotics, irrational use (incorrect dosage) or improper use can cause public health problems such as the presence of antibiotic residues in food intended for human consumption, which is dangerous for human health. Resistance to fish bacteria can be transmitted to other terrestrial animals, humans and the environment in general, with reduction of the drug's therapeutic potential.

A lack of legislation and regulations on antibiotics promotes not only illicit acquisition but also self-medication by farmers [37], which leads to intensive and unnecessary use of antibiotics in fish farming. Due to the nonbiodegradability of most antibiotics used [38], their intensive and unnecessary use in fish farming results in the development and spread of resistant bacteria and the presence of antimicrobial residues in the environment, which represent dangerous risks to public health [6, 39]. The consequences of the use of antimicrobials in fish farms include the deposition of residues in muscles designated for human consumption irrespective of the route or purpose of administration before they are completely metabolised or excreted from the body [40]. The relative stability and nonbiodegradation of some antibiotics accounts for the presence of residues in commercial fish [7].

The level of acceptable attitudes towards AMU was poor in the study area, which contrasts with the high percentage of favourable attitudes (72.22%) obtained by Waga et al. in 2022 in Nairobi, Kenya [30]. The level of education, the training of fish farmers and the presence of animal health agents can be attributed to this low score. Additionally, the attitude score towards AMU increased with the level of education. In addition, the score was significantly higher on farms with a health worker and among those trained in fish farming. The strong positive correlation between attitudes towards AMU and knowledge about AMU, on the one hand, and between attitudes towards AMU and knowledge about AMR, on the other hand, explicitly justifies the need to promote learning about antibiotic use and AMR to achieve acceptable attitudes towards antibiotic use in the face of the dangers that misuse of antibiotics constitutes for public health.

The practice of antibiotic use was poor and comparable to that reported by Waga eta l. [30], who reported a low percentage of adequate AMU practices (33.33%) in Kenya. This result could be explained by the lack of involvement of health workers, the lack of training in fish farming, the farming system, the level of education and the age of the fish farmers. Once again, this score calls for the collective conscience of fish farmers to leave the handling of drugs to health professionals and to train them in fish farming. If the level of education of the latter is also called into question, it should be mentioned that promoting the intensification of fish farming in Cameroon would be an asset for good practices in the use of antibiotics. Younger fish farmers should be encouraged because they are better able to adapt to new technologies. The positive correlations between the antibiotic use practices and AMU knowledge, and between AMR knowledge and the attitudes suggests a strong association between these variables. Although this relationship does not imply causality, it highlights that higher levels of knowledge and more favourable attitudes were generally associated with more appropriate practices. This finding underscores the potential of cognitive and behavioural components in promoting the rational use of antimicrobials. However, further research, particularly longitudinal or interventional studies, is needed to better understand the directionality and nature of this association. This evidence is reinforced by the fact that more than half and almost three-quarters of the surveyed fish farmers are unaware of the problems associated with the misuse of antibiotics and practices that can induce resistance to antibiotics, respectively. Though increased specific trainings, involvement of health professionals, and other awareness-raising methods were observed, the rational use of antimicrobials among fish farmers and the associated financial and logistical challenges remained major constraint factors. The finding agrees with Patil et al. [41], who observed in India that although fish farmers showed acceptable levels of awareness and understanding for the use of antimicrobials, proper implementation of rational antimicrobial use practices was often hindered by logistical, financial, and accessibility issues. In the present study, a high response rate was achieved due to a data collection strategy based on in-person participation. The questionnaire allowed for the collection of detailed information on farmers' behaviour regarding AMU and AMR, as well as their practices, attitudes and risk perceptions. However, the identified behavioural and farm-related factors do not explain the complete variance observed in each category.

Despite the low level of knowledge of fish farmers regarding AMU and AMR, practices and attitudes, fish farmers perceive the emergence of AMR as a risk to public health. Therefore, increasing awareness among farmers during awareness campaigns or developing farmer-friendly educational materials that effectively communicate the risks of AMR in aquaculture can help to fill the gaps in knowledge and support alternative disease prevention and management strategies to the use of antimicrobial agents to address hygiene adulteration.

## 5. Conclusions

This study highlights low levels of knowledge of fish farmers regarding antimicrobial use, and resistance, practices and attitudes toward antimicrobial use and perceptions of the risk of AMR emergence in the study regions. Consequently, this implies a change in behaviour by improving the knowledge of the various stakeholders in the fish farming sector about AMU. Indeed, knowledge is a useful predictor of behavioural changes and can enhance the ability to adopt rational AMU. The sensitisation of fish farmers and other stakeholders to proper AMU and alternatives is important for reducing the risk of the emergence of resistant bacteria and their transmission to public health in Cameroon. This study highlights the importance to strengthen regulatory frameworks governing the use, consumption and distribution of antimicrobials. Further work is essential to assess the challenges and opportunities related to the use and impacts of antimicrobials in fish farming and awareness tools for the rational use of antimicrobials and AMR.

## Figures and Tables

**Table 1:** Geographical and demographic characteristics of the study areas.

Characteristics	Region				
	Adamawa	Centre	Littoral	South	West
Geographical coordinates	6°10′–7°90′LN	3°52′–6°14′LN	4°03′–4°90′LN	2°55′–12.00″LN	5°02′–5°39′LN
	11°10′–15°60′LE	11°31′–12°93′LE	9°42′–10°43′LE	11°38′-56.40″LE	9°40′–11°11′LE

Area (km^2^)	63701	68926	20249	47720	13892

Climate	Sudano-guinean	Equatoguinean	Equatorial	Equatorial	Equatoguinean

Population	1,200,970	4,159,500	3,354,978	749,552	2,770,400

Density (hab/km^2^)	18	60	170	16	200

Annual rainfall (mm)	25	1643	3702	3250	1871

Annual temperature (°C)	23.5	23.7	26.2	25	20

Attitude (m)	1100	750	940	450	1390

**Table 2:** Demographics and farm characteristics of the surveyed fish farms (*N* = 364) in the Adamawa, Centre, Littoral, South and West regions of Cameroon.

Factors	Variables	Respondents, *n* (% [95% CI])
Region	Adamawa	34 (9.34 [6.76–12.77])
	Centre	110 (30.22 [25.73–35.12])
	Littoral	103 (28.30 [23.91–33.13])
	South	22 (6.04 [4.02–8.98])
	West	95 (26.09 [21.85–30.84])

Gender	Male	273 (75.00 [70.31–79.17])
	Female	91 (25.00 [20.83–29.69])

Age range (years)	Less than 25	16 (4.40 [2.72–7.02])
	26–35	147 (40.38 [35.47–45.50])
	36–50	149 (40.93 [36.00–46.05])
	More 50	52 (14.29 [11.06–18.25])

Level of education	None	9 (2.47 [1.31–4.63])
	Primary	34 (9.34 [6.76–12.77])
	Secondary	105 (28.85 [24.43–33.70])
	Higher	216 (59.34 [54.22–64.26])

Training in fish-farming	Yes	299 (82.14 [77.88–85.74])
	No	65 (17.86 [14.26–22.12])

Fish-farming as sources of income	Yes	98 (26.92 [22.62–31.70])
	No	266 (73.07 [68.30–77.38])

Experience in fish-farming	Less than 5 years	258 (70.89 [66.01–75.31])
	5–10 years	72 (19.78 [16.01–24.18])
	More than 10 years	34 (9.34 [6.76–12.77])

Type of farm	Cooperative	30 (8.24 [5.83–11.52])
	Common initiative group	88 (24.18 [20.06–28.83])
	Family exploitation	219 (60.16 [55.05–65.06])
	Others^∗^	27 (7.42 [5.15–10.58])

Husbandry system	Extensive	40 (10.99 [8.17–14.62])
	Semiextensive	15 (4.12 [2.51–6.69])
	Intensive	309 (84.89 [80.85–88.20])

Consult animal health personnel	Yes	158 (43.41 [38.41–48.54])
	No	206 (56.59 [51.46–61.59])

Fish load	0–1000	68 (18.68 [15.01–23.00])
	1001–2000	77 (21.15 [17.27–25.64])
	2001–3000	71 (19.51 [15.76–23.89])
	3001–4000	32 (8.79 [6.30–12.15])
	4001–5000	34 (9.34 [6.76–12.77])
	5001–6000	13 (3.57 [2.10–6.01])
	6001–10000	34 (9.34 [6.76–12.77])
	10001–15000	13 (3.57 [2.10–6.01]))
	More than 15000	22 (6.04 [4.02–8.98]))

Production cycle (month)	Less than 8	275 (75.55 [70.88–79.68]))
	8–12	65 (17.86 [14.26–22.12]))
	More than 12	24 (6.59 [4.47–9.62]))

Biosecurity level	Poor	46 (12.64 [9.61–16.45]))
	Fair	304 (83.51 [79.36–86.97]))
	Good	14 (3.85 [2.30–6.35]))

^∗^Others include personal exploitation, experimental farms, establishment and limited liability companies.

**Table 3:** Evaluation of basic knowledge about antibiotics among fish farmers.

Variables	Number of respondents	Proportion % [95% CI]
Heard about AB	364	
No	44	12.09 [9.13–15.84]
Yes	320	87.91 [84.56–91.26]

Definition of AB	320	
Good answer	241	75.31 [70.31–79.72]
Wrong answer	79	24.69 [20.28–29.69]

Germs on which Abs are effective	320	
Bacteria	224	70.00 [64.77–74.76]
Fungi	72	22.50 [18.27–27.39]
Virus	24	7.50 [5.09–10.92]
Parasite	78	24.38 [19.99–29.36]
I don't know	140	43.75 [38.42–49.23]
Others	3	0.94 [0.32–2.72]

^∗^Others include pain, stress and wounds.

Abbreviation: AB = antibiotic.

**Table 4:** Distribution of mean knowledge scores of AMU and AMR versus demographic characteristics and farm characteristics of fish farmers (*n* = 320) in the Adamawa, Centre, Littoral, South and West regions of Cameroon.

		AMU mean ± SD	*p* value	AMR mean ± SD	*p* value
Region	Adamawa	0.46^ab^ ± 0.23		0.31^ab^ ± 0.25	
	Centre	0.43^b^ ± 0.18		0.24^b^ ± 0.30	
	Littoral	0.53^a^ ± 0.18	0.002^∗∗^	0.44^a^ ± 0.32	< 0.001^∗∗∗^
	South	0.47^ab^ ± 0.18		0.24^b^ ± 0.26	
	West	0.47^ab^ ± 0.20		0.24^b^ ± 0.22	

Gender	Male	0.48 ± 0.19		0.31 ± 0.29	
	Female	0.46 ± 0.20	0.234	0.30 ± 0.28	0.815

Age of manager	Less than 25	0.54 ± 0.22		0.36 ± 0.26	
	26–35	0.48 ± 0.19	0.053	0.33 ± 0.30	0.139
	36–50	0.47 ± 0.20		0.28 ± 0.29	
	More than 50	0.41 ± 0.16		0.25 ± 0.28	
	None	0.35^b^ ± 0.15		0.16^b^ ± 0.23	

Educational level	Primary	0.41^b^ ± 0.20	< 0.001^∗∗∗^	0.17^b^ ± 0.23	0.001^∗∗^
	Secondary	0.42^b^ ± 0.18		0.26^ab^ ± 0.27	
	Higher	0.51^a^ ± 0.19		0.35^a^ ± 0.30	

Professional experience	Less than 5	0.46 ± 0.19		0.30 ± 0.30	
	5–10	0.49 ± 0.20	0.729	0.32 ± 0.28	0.572
	More than 10	0.48 ± 0.20		0.27 ± 0.26	

Husbandry system	Extensive	0.43 ± 0.18		0.22 ± 0.26	
	Semiextensive	0.48 ± 0.19	0.218	0.31 ± 0.29	0.124
	Intensive	0.49 ± 0.24		0.34 ± 0.31	

Farm as principal activity	Yes	0.49 ± 0.19		0.32 ± 0.31	
	No	0.47 ± 0.19	0.338	0.30 ± 0.29	0.575

Training in fish-farming	Yes	0.49 ± 0.20		0.33 ± 0.29	
	No	0.39 ± 0.15	< 0.001^∗∗∗^	0.20 ± 0.25	0.001^∗∗^

Presence of heath agent	Yes	0.50 ± 0.20		0.31 ± 0.29	
	No	0.45 ± 0.19	0.021^∗^	0.30 ± 0.29	0.986
	Total	0.47 ± 0.19		0.30 ± 0.29	

^a^, ^b^, ^ab^: Pairwise comparisons between the groups.

^∗^ *p* < 0.05; ^∗∗^*p* < 0.01; ^∗∗∗^*p* < 0.001.

**Table 5:** Distribution of the mean scores of attitudes, practices and risk perceptions of antimicrobial resistance versus demographic characteristics and farm characteristics of fish farmers (*n* = 320) in the Adamawa, Centre, Littoral, South and West regions of Cameroon.

		Attitude	*p* value	Practices	*p* value	Perception	*p* value
		Mean ± SD		Mean ± SD		Mean ± SD	
Region	Adamawa	0.38 ± 0.31		0.48^ab^ ± 0.13		0.59^a^ ± 0.32	
	Centre	0.39 ± 0.31		0.50^a^ ± 0.10		0.52^a^ ± 0.26	
	Littoral	0.44 ± 0.26	0.087	0.45^b^ ± 0.10	0.001^∗∗^	0.50^ab^ ± 0.30	0.005^∗∗^
	South	0.30 ± 0.25		0.42^b^ ± 0.09		0.33^b^ ± 0.28	
	West	0.39 ± 0.28		0.48^ab^ ± 0.12		0.50^ab^ ± 0.28	

Gender	Male	0.40 ± 0.28		0.47 ± 0.11		0.50 ± 0.29	
	Female	0.39 ± 0.29	0.662	0.48 ± 0.11	0.676	0.50 ± 0.29	0.899

Age of manager	Less than 25	0.39 ± 0.28		0.52^a^ ± 0.10		0.49 ± 0.29	
	26–35	0.42 ± 0.28	0.508	0.49^ab^ ± 0.11	< 0.001^∗∗∗^	0.51 ± 0.29	0.811
	36–50	0.38 ± 0.30		0.47^ab^ ± 0.11		0.49 ± 0.29	
	More than 50	0.38 ± 0.26		0.42^b^ ± 0.09		0.53 ± 0.27	

Educational level	None	0.17^b^ ± 0.20		0.46^b^ ± 0.12		0.30^b^ ± 0.24	
	Primary	0.32^b^ ± 0.31	< 0.001^∗∗∗^	0.45^b^ ± 0.10	0.018^∗^	0.50^a^ ± 0.27	0.009^∗∗^
	Secondary	0.31^b^ ± 0.26		0.45^b^ ± 0.11		0.46^ab^ ± 0.30	
	Higher	0.46^a^ ± 0.28		0.49^a^ ± 0.11		0.54^a^ ± 0.28	

Professional experience	Less than 5	0.38 ± 0.29		0.47 ± 0.11		0.47^a^ ± 0.29	
	5–10	0.43 ± 0.28	0.546	0.50 ± 0.12	0.077	0.57^b^ ± 0.29	0.006^∗∗^
	More than 10	0.38 ± 0.29		0.45 ± 0.12		0.58^b^ ± 0.26	

Husbandry system	Extensive	0.33 ± 0.28		0.43^b^ ± 0.11		0.47 ± 0.25	
	Semiextensive	0.41 ± 0.28	0.261	0.48^a^ ± 0.11	0.008^∗∗^	0.51 ± 0.29	0.617
	Intensive	0.42 ± 0.29		0.51^a^ ± 0.14		0.55 ± 0.33	

Farm as principal activity	Yes	0.44 ± 0.28		0.48 ± 0.12		0.53 ± 0.28	
	No	0.39 ± 0.28	0.106	0.47 ± 0.11	0.440	0.49 ± 0.29	0.218

Training in fish-farming	Yes	0.43 ± 0.28		0.48 ± 0.11		0.53 ± 0.29	
	No	0.28 ± 0.27	< 0.001^∗∗∗^	0.45 ± 0.09	0.036^∗^	0.40 ± 0.26	0.001^∗∗∗^

Presence of heath agent	Yes	0.44 ± 0.27		0.50 ± 0.12		0.53 ± 0.28	
	No	0.37 ± 0.29	0.024^∗^	0.45 ± 0.10	< 0.001^∗∗∗^	0.48 ± 0.29	0.089
	Total	0.40 ± 0.28		0.47 ± 0.11		0.50 ± 0.29	

Abbreviations: AMR = antimicrobial resistance; AMU = antimicrobial use.

^a^, ^b^, ^ab^: Pairwise comparison between the groups.

^∗^ *p* < 0.05; ^∗∗^*p* < 0.01; ^∗∗∗^*p* < 0.001.

**Table 6:** Pearson's correlation coefficients for fish farmers (*N* = 320) in the Adamawa, Centre, Littoral, South and West regions of Cameroon.

	Variable	K-AMU	K-AMR	Attitude	Practice	Perception	*N*
Pooled	K-AMU	—					364
	K-AMR	0.63^∗∗∗^	—				
	Attitude	0.62^∗∗∗^	0.72^∗∗∗^	—			
	Practice	0.33^∗∗∗^	0.20^∗∗∗^	0.29^∗∗∗^	—		
	Perception	0.48^∗∗∗^	0.55^∗∗∗^	0.60^∗∗∗^	0.27^∗∗∗^	—	
	Biosecurity	0.17^∗∗^	0.09	0.20^∗∗∗^	0.35^∗∗∗^	0.24^∗∗∗^	

Adamawa	K-AMU	—					34
	K-AMR	0.72^∗∗∗^	—				
	Attitude	0.82^∗∗∗^	0.84^∗∗∗^	—			
	Practice	0.22	0.29	0.17	—		
	Perception	0.76^∗∗∗^	0.80^∗∗∗^	0.85^∗∗∗^	0.20	—	
	Biosecurity	0.58^∗∗∗^	0.58^∗∗∗^	0.62^∗∗∗^	0.51^∗∗^	0.64^∗∗∗^	

Centre	K-AMU	—					110
	K-AMR	0.72^∗∗∗^	—				
	Attitude	0.61^∗∗∗^	0.77^∗∗∗^	—			
	Practice	0.44^∗∗∗^	0.29^∗∗^	0.26	—		
	Perception	0.63^∗∗∗^	0.67^∗∗∗^	0.68^∗∗∗^	0.30^∗∗^	—	
	Biosecurity	0.32^∗∗∗^	0.19^∗^	0.19^∗^	0.36^∗∗^	0.30^∗∗^	

Littoral	K-AMU	—					103
	K-AMR	0.57^∗∗∗^	—				
	Attitude	0.57^∗∗∗^	0.73^∗∗∗^	—			
	Practice	0.27^∗∗^	0.16^∗∗^	0.31^∗∗^	—		
	Perception	0.53^∗∗^	0.63^∗∗∗^	0.72^∗∗∗^	0.44^∗∗^	—	
	Biosecurity	0.22^∗∗^	0.17^∗∗^	0.33^∗∗^	0.35^∗∗^	0.36^∗∗^	

South	K-AMU	—					22
	K-AMR	0.58^∗∗^	—				
	Attitude	0.50^∗^	0.78^∗∗∗^	—			
	Practice	−0.07	−0.28	−0.14			
	Perception	0.22	0.45^∗^	0.47^∗^	0.30	—	
	Biosecurity	0.05	0.06	0.02	0.05	0.14	

West	K-AMU	—					95
	K-AMR	0.57^∗∗^	—				
	Attitude	0.63^∗∗∗^	0.66^∗∗∗^	—			
	Practice	0.55^∗∗^	0.46^∗∗^	0.46^∗∗^	—		
	Perception	0.31^∗^	0.35^∗^	0.31^∗^	0.05	—	
	Biosecurity	0.10	0.06	0.06	0.18	−0.08	

Abbreviations: K-AMR = knowledge on antimicrobial resistance; K-AMU = knowledge on antimicrobial use.

^∗^ *p* < 0.05; ^∗∗^*p* < 0.01; ^∗∗∗^*p* < 0.001.

**Table 7:** Linear regression model of knowledge, attitudes, practices and risk perception measures of fish farmers according to demographic characteristics (*N* = 320) and farm characteristics in the Adamawa, Centre, Littoral, South and West regions of Cameroon.

Variable		Knowledge of AMU	Knowledge of AMR	Attitudes	Practices	Perceptions
Demographic characteristic	Region	0.013^∗^	0.016	0.010	−0.008	−0.017
	Gender	0.022	0.005	−0.004	−0.003	−0.026
	Age of responsible	−0.022^∗^	−0.035	−0.012	−0.036^∗∗^	−0.008
	Experience	0.015	−0.040	0.008	0.020^∗^	0.070^∗∗^
	Educational level	−0.025^∗∗^	−0.032	−0.053^∗∗^	−0.008	−0.018
	Training in fish-farming	0.076^∗∗∗^	0.113^∗∗^	0.115^∗∗^	0.0003	0.092^∗^
	Farm as principal activity	0.014	0.004	−0.012	0.005	−0.016
	Presence of health agent	0.034^∗^	0.003]	0.063^∗^	0.045^∗∗^	0.034
	Constant	0.171^∗^	0.215	0.262	0.531^∗∗^	0.378^∗^
		*R* ^2^ = 0.110; *F* = 6.89^∗∗^	*R* ^2^ = 0.052; *F* = 5.69^∗∗^	*R* ^2^ = 0.085; *F* = 5.09^∗∗^	*R* ^2^ = 0.124; *F* = 5.52^∗∗^	*R* ^2^ = 0.066; *F* = 2.30^∗^

Zootechnical characteristic	Fish density	−0.001	−0.001	0.002	0.004	−0.001
	Duration of breeding cycle	0.006	−0.047	0.012	0.016	−0.016
	Age of exploitation	0.002	−0.010	−0.004	−0.007	0.034^∗∗^
	Husbandry system	0.008	0.001	0.018	0.003	0.002
	Total biosecurity	0.082	0.248	0.314^∗^	0.190^∗∗^	0.267^∗^
	Constant	0.2662^∗∗∗^	0.334^∗∗^	0.199	0.349^∗∗^	0.290^∗^
		*R* ^2^ = 0.035; *F* = 1.89	*R* ^2^ = 0.026; *F* = 1.45	*R* ^2^ = 0.039; *F* = 2.85^∗^	*R* ^2^ = 0.140; *F* = 9.78^∗∗^	*R* ^2^ = 0.089; *F* = 1.84^∗^

Abbreviations: AMR = antimicrobial resistance; AMU = antimicrobial use.

^∗^ *p* < 0.05; ^∗∗^*p* < 0.01; ^∗∗∗^*p* < 0.001.

**Table 8:** Distribution of fish farmers (*N* = 320) according to KAPP level in the Adamawa, Centre, Littoral, South and West regions of Cameroon.

KAPP level	KAMU *n* (%) [95% CI]	KAMR *n* (%) [95% CI]	Attitude *n* (%) [95% CI]	Practice *n* (%) [95% CI]	Perception *n* (%) [95% CI]
Poor	234 (73.13) [68.01–77.69]	231 (72.19) [67.04–76.81]	204 (63.75) [58.35–68.83]	254 (79.38) [74.61–83.45]	211 (65.94) [60.58–70.91]
Fair	10 (3.13) [1.71–5.66]	16 (5.00) [3.1–7.97]	46 (14.38) [10.95–18.64]	0	0
Good	76 (23.75) [19.42–28.71]	73 (22.81) [18.55–27.72]	70 (21.88) [17.69–26.72]	66 (20.63) [16.55–25.39]	109 (34.06) [29.09–39.42]

Abbreviations: K-AMR = knowledge on antimicrobial resistance; K-AMU = knowledge on antimicrobial use.

## Data Availability

The data that support the findings of this study are available from the corresponding author upon reasonable request.
